# SURC (Symptom and Urgent Review Clinic) in oncology practice: clinical utility and application in a regional setting—a retrospective observational study

**DOI:** 10.1007/s00520-026-10629-7

**Published:** 2026-04-30

**Authors:** Suzanne Bartlett, Armand Gumera, Michael Mil, Stephen Brown, Dan Stout, Wasek Faisal

**Affiliations:** 1 Department of Medical Oncology, BRICC, Grampians Health, Victoria, Australia; 2 University of Melbourne, Parkville, Australia; 3 La Trobe University, Melbourne, Australia

**Keywords:** Symptom and urgent review clinic (SURC), Patient-centred oncology care, Regional healthcare delivery, Healthcare resource optimisation

## Abstract

**Purpose:**

To assess the benefit of the new ‘SURC’ model, allowing patients having systemic anti-cancer therapy (SACT) to be seen acutely for assessment of adverse events, as well as scheduled early review in their treatment course. It also assessed the effect of the COVID-19 pandemic on this service.

**Methods:**

Data was collected prospectively between 2020 and 2023 and then analysed retrospectively. The clinic was implemented at Ballarat Regional Integrated Cancer Centre (BRICC). It also included two outreach sites: Primary outcomes were the number of interactions by patients with SURC; secondary outcomes included the number of admissions, type of cancer involved, and reasons for contacting SURC.

**Results:**

This study demonstrated benefit for patients with cancer from a regional area who presented with acute complications of their treatment. Presentations requiring hospital admissions and further assessment in ED were lower compared to the pre-SURC period, with most patients being treated comprehensively in the SURC environment. The study demonstrated the utility of early SACT review as there was a decrease in the number of treatment disruptions due to early recognition of complications of each patient’s regimen. There was also a gradual increase in patient presentations to the clinic during the duration of the study, reflecting the growing awareness and confidence in the service from both healthcare providers and patients alike.

**Conclusion:**

This study demonstrates the benefit of the novel SURC model to patients living in regional areas with cancer who either develop acute complications or are routinely reviewed early during their course of treatment. Although this study was limited to only one public health service provider, we feel this new proactive paradigm for acute cancer care will become increasingly adopted across Australia, given its proven ability to look after patients undergoing SACT more efficiently in addition to reducing hospital admissions.

## Relevance statement

1. Patients with cancer having systemic therapy regularly develop complications that require prompt review, and benefit from specialist oncology review both for acute symptoms and for assessment early in their treatment. Patients in regional areas also have more limited access to these services.
2. This study demonstrates the benefit of a dedicated model for early review and assessment for patients with cancer and their associated complications of treatment. It also gave a unique insight into looking after patients during the COVID-19 pandemic.
3. This study supports further establishing the SURC model, particularly in non-metropolitan areas.

## Introduction

The complex sequelae of symptoms arising from cancer and systemic anti-cancer therapies require early recognition and prompt, thorough management [1, 2]. Responding to these complications in a timely manner is necessary to prevent further complications, treatment delays, and hospital admissions, often via the emergency department (ED). For patients who live outside of a metropolitan area, the demands of treatment are exacerbated by a dearth of local specialised resources [3] and if presentation to hospital is required, it often involves a long commute. Non-urgent presentations to ED for patients receiving anticancer therapy are also becoming increasingly prevalent across all healthcare regions [4]. There is a demonstrable need for a service that can meet the needs of this large patient group [5].

The Symptom and Urgent Review Clinic (SURC) model was initially introduced as a response to the demands of acute complications of systemic anti-cancer treatment (SACT) in Australia in 2013. This pilot model was based in the Western Health public health service region, Victoria [6]. A key feature of the SURC model is its shared care approach, which involves a multidisciplinary team of oncology nurses, oncologists, and supportive care staff [7] 

Since inception, the SURC model has been implemented in many hospitals across Victoria, providing acute access to oncology advice for patients who encounter complications due to cancer or related treatments. The clinics are operated by senior oncology nurses who offer in-person and telehealth reviews, serving as a continuous point of contact for patients.

Given the limited published data on the implementation and impact of the SURC model, our study was designed to give increased insight into a novel regional model. A detailed review of such a service is important not only for reviewing direct patient care in the more remote setting, but to assess its role and benefit within the national healthcare system.

The aim of this study was to assess the utility and uptake of SURC in managing patients undergoing anti-cancer treatments (i.e. chemo/immunotherapy, oral targeted therapies, endocrine treatment, supportive care) at the Ballarat Regional Integrated Cancer Centre (BRICC), a large, regional, tertiary cancer service in Victoria, as well as patients from Maryborough District Health Service (MDHS) and Stawell Regional Health (SRH), two sites that BRICC provides an oncology outreach service to. Collectively, the BRICC SURC provides a service to an approximately 50,000 km^2^ catchment area across these three sites, which is uniquely challenging, given both the size of the region and the complexity of medical issues that patients often present with.

We assessed two primary outcomes in this study, the volume and nature of presentations, and the reasons for patient presentation to SURC. By analysing data from all presentations of BRICC patients to SURC between 1 May 2020 and 30 April 2023, we accumulated insights into the evolution of patient care in an acute, oncological context, and the value that the SURC model brings with its multidisciplinary healthcare approach.

## Methods

This was a retrospective observational study of all oncology patients that had contact with SURC between 1 May 2020 and 30 April 2023. All episodes of care, including treatment education, phone, and in-person attendances, were included. Data collection involved recording information fields such as tumour type, treating unit (haematology or oncology), and patient location (primary or outreach). ED presentations of Grampians Health patients were recorded to assess whether SURC reduced such presentations. Patient experience surveys were conducted post-SURC phone contact or in-person. Statistical analyses were performed to describe the data, test for associations, correlations, and statistical significance. Descriptive statistics were performed in SPSS version 28. Comparisons were made with the chi-squared test or Fisher’s exact test for categorical variables, and the independent *t*-test or ANOVA for numerical variables, depending on the data variable. The significance level was set at *P* < 0.05. Due to the non-identifiable nature of the data, and that no demographic information was collected, formal ethics approval was waived by the Grampians Health ethics committee.

### The SURC model at BRICC

The objective of SURC is to recognise and manage symptoms from cancer and cancer-related treatment early, reducing the need for ED visits, hospital admissions or treatment delays. SURC involves a multidisciplinary team comprising of oncology nurses, oncologists, and supportive care staff. The clinic operates within a dedicated space equipped with examination rooms, consultation areas, and treatment bays with necessary medical equipment. The physical layout is designed to ensure patient privacy during consultations and assessments. On first contact with patients, they are triaged, and appropriate review organised. The triage process involves a telephone assessment by experienced oncology nurses to determine the urgency and severity of symptoms using an evidenced-based Telephone Triage Tool launched by eviQ in 2019. This tool was initially developed by UKONS [8] but adjusted to be used and embedded in Australia. Patient reviews are performed either in person or via telehealth. Alongside this urgent review service, the SURC team also facilitates routine early treatment reviews, as well as being a first point of contact for patients with cancer who contract COVID-19, to facilitate early assessment, prescription of antivirals, follow-up care and coordinating with the treating team to reschedule their subsequent SACT and clinic appointments.

## Results

### Volume and nature of presentations

SURC data collection commenced in May 2020 at the inception of the clinic. For calculations per month, our analysis was calculated from the last 8 months of 2020, the 24 months of 2021 and 2022, and the first 4 months of 2023.

In 2018, 1682 oncology patients presented to the Emergency Department at our health service. Of this total number, 611 patients were able to be discharged home, 47 patients left at their own risk, and another 145 patients were admitted in the short-stay unit.

The total patient presentations to SURC rose from 1026 in 2020 (128 per month from May to December 2020), to 1825 in 2021 (228 per month), to 2866 in 2022 (239 per month), and then 1194 in the first 4 months of 2023 (299 per month, *P* < 0.001). The increased number of presentations in 2020 may have been due to the temporary cessation of in-person elective care at hospitals due to the evolving pandemic at the time.

The total number of episodes of care over the study period (*n* = 6911) consisted of SURC clinic reviews not requiring hospital admission (*n* = 834; 12%), same-day admissions (*n* = 272; 4%), telephone calls (*n* = 5490; 79%), and ED admissions that could have presented to SURC (*n* = 315; 5%). In 2020, the proportion of non-urgent ED presentations was 5.1%. In 2021, this increased to 5.9% and then dropped down to 3.7% in 2022 and 4.0% in 2023 (*P* < 0.001). The increase in ED presentations in 2021 was thought to be due to patients who contacted SURC with symptoms of COVID-19 having to be instead referred to ED as directed by health service guidelines. Data showed a reduction in the proportion of patients going to ED by 22% from 2020 to 2023. This suggests that SURC decreased the rate of non-urgent ED presentations, streamlining patient care and leading to improved healthcare resource allocation.

**Fig. 1:**
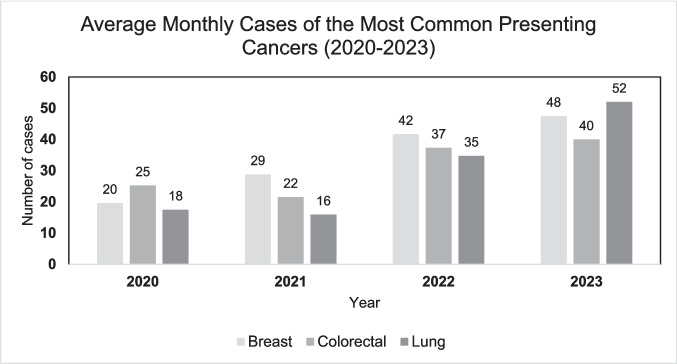
Comparison of SURC service uptake across different common cancer types, demonstrating an overall increase in utilisation

**Fig. 2:**
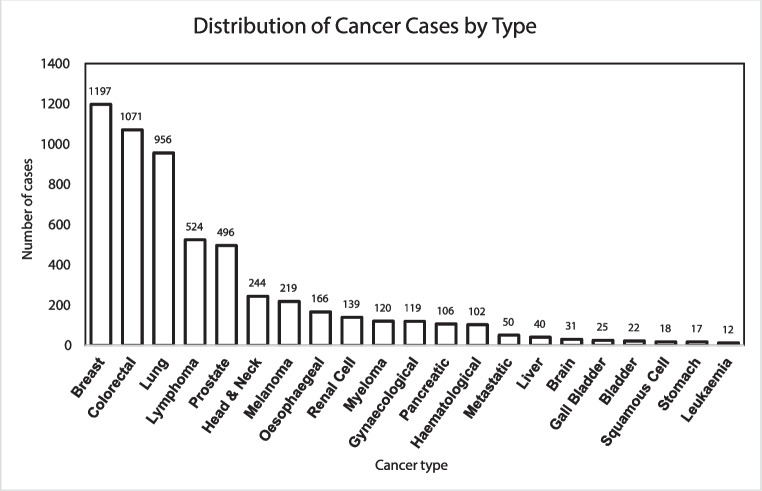
Column graph of the most commonly presenting cancer types shows that breast cancer (1197 cases) and colorectal cancer (1071 cases) were the most frequently diagnosed, followed by lung cancer (956 cases). Notably, lymphoma (524 cases), prostate cancer (496 cases), and melanoma (244 cases) also demonstrated substantial representation

The three most common tumour streams that patients have systemic anti-cancer treatment for at BRICC are breast, colorectal, and lung cancer (Fig. 1). These higher rates of presentation for these tumour streams were mirrored in the data on patients that presented to SURC; however, there were patients with cancer from many different types of tumour stream presenting in this period (Fig. 2).

Over the study period, the most prevalent tumour streams for patients were breast cancer (1197), colorectal cancer (1071), and lung cancer (956). In 2020, there were 157 breast cancer, 202 colorectal cancer, and 140 lung cancer presentations. In 2021, the number of breast cancer cases increased to 346, colorectal cancer cases to 259, and lung cancer cases to 191. In 2022, there was a further increase in all three again: breast cancer (500), colorectal cancer (448), and lung cancer (417) presentations. Finally, in the first 4 months of 2023, there were 190 breast, 160 colorectal, and 208 lung cancer cases.

Analysis per month demonstrated a steady increase in presentations (Table 1). The consistently high presentation of patients with these three cancer types suggests that SURC is likely playing an essential role in managing their care and preventing higher rates of ED presentation and hospital admissions. For this patient population, there are multiple possible treatment regimens including chemotherapy, immunotherapy, radiotherapy, and targeted therapies. Any of these regimens may cause multiple symptoms and toxicities, leading to a high number of SURC presentations. This increase overlaps with the pandemic, which saw a shift towards remote patient monitoring and telehealth. SURC’s integrated telephone consultation service demonstrated its important role during this period, allowing for continued care amidst social distancing measures. Its role in early symptom recognition minimised non-urgent hospital visits, reducing potential COVID-19 exposure for vulnerable patients.

**Table 1:** The total number of interactions per year across health care services, ED admissions as a percentage of that year, total number of interaction types across all four years, and total interaction type as a percentage of yearly total

	All health services				Total per interaction	Interactions % of yearly total
	2020	2021	2022	2023		
Telephone calls	766	1390	2349	985	5490	79%
Clinic presentations	142	263	309	120	834	12%
Same-day admissions	66	64	101	41	272	4%
ED admissions	52	108	107	48	315	5%
Yearly total	1026	1825	2866	1194	6911	
Interactions per month	102.6	152.1	238.8	298.5		
% ED admissions	5.1%	5.9%	3.7%	4.0%		

Furthermore, by diverting non-emergency cases away from the ED, SURC assisted in the enhanced allocation of resources during a period where healthcare systems were under significant strain.

Most interactions over the study period consisted of telephone consultations. This number increased from 766 (96 per month) in 2020, to 1390 (116 per month) in 2021, 2349 (196 per month) in 2022, and then to 985 (246 per month) in 2023 (*P* < 0.001). The number of same-day admissions after the initial phone call varied from 66 (8 per month) in 2020, to 64 (5 per month) in 2021, 101 (8 per month) in 2022, and 41 (10 per month) in 2023, but this was not of statistical significance (*P* = 0.65). As the overall numbers of SURC presentations per month increased, non-urgent ED admissions also grew, from 52 (7 per month) in 2020, to 108 (9 per month) in 2021, to 107 (9 per month) in 2022, and 48 (12 per month) in 2023 (*P* = 0.045).

#### Admissions through SURC

The data from 2020 shows there were 8 same-day admissions per month from SURC. This figure increased to 10 per month in 2023. This suggests that SURC is increasingly being used as a direct route for hospital admissions when necessary.

#### Admissions through ED

Emergency Department admissions followed a slightly different trend. There were 7 per month in 2020, which increased to 12 per month in 2023. This increase could have been caused by the inclusion of 2022 and 2023 data from Maryborough and Stawell outreach services. While these numbers represent a small proportion of total cases, our data suggests that SURC can potentially help to prevent non-urgent ED admissions by addressing health issues promptly and effectively without affecting ED resources (Table 2).

**Table 2:** The total admissions across all four years, total number of patients managed without hospital admission, and the monthly average of this. These differences were statistically significant (*P* < 0.001)

	2020	2021	2022	2023
Admissions total (ED + same-day)	118	172	208	89
Yearly total	1026	1825	2866	1194
Managed without hospital Admission	908	1653	2658	1105
Monthly average	114	138	222	276

#### Patients managed without hospital admission

The number of patients who had consultations via SURC but did not require hospital admission (same-day or via ED) was calculated by subtracting the total number of ED and same-day admissions from the number of total presentations. The number of patients managed without hospital admission increased from 908 (114 per month) in 2021 to 1653 (138 per month) in 2021, to 2658 (222 per month) in 2022, and finally to 1105 (276 per month) in 2023 (*P* < 0.001).

### Reasons for patient contact with SURC

**Fig. 3:**
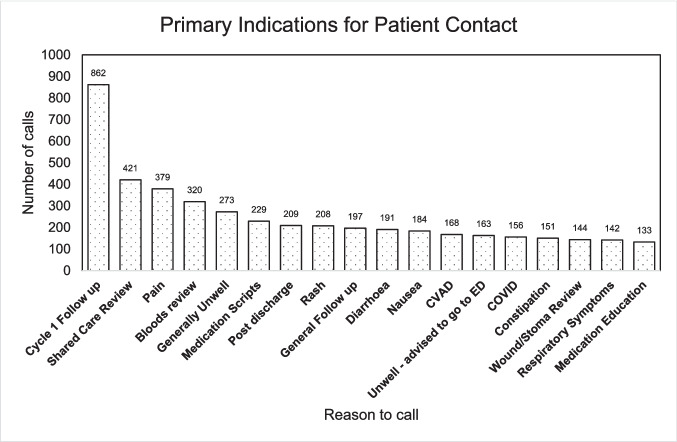
Column graph showing the number of calls to SURC per reason. Cycle 1 follow-up was the most common reason, comprising 862 calls, which represented 11% of total calls. There were 7808 telephone calls in total across the study period. CVAD, central venous access devices

Patients contacted SURC at Grampians Health for a variety of reasons (Fig. 3). These reasons can be grouped into five broad categories:

- Symptoms and health concerns: This category includes symptoms directly related to cancer or its treatment such as pain (*n* = 379), abdominal pain (*n* = 41), diarrhoea (*n* = 191), nausea (*n* = 184), vomiting (*n* = 52), respiratory symptoms (*n* = 142), neurological symptoms (*n* = 66), rash (*n* = 208), and febrile symptoms (*n* = 18). It also includes symptoms not directly linked to cancer but may be side effects of the treatment or indicative of other health issues such as fatigue (*n* = 58), dizziness (*n* = 23), headaches (*n* = 23), hypertension (*n* = 14), and insomnia (*n* = 4).
- Medication and treatment queries: This includes queries related to medication scripts (*n* = 229), cytotoxic management (*n* = 1), electrolyte replacement (*n* = 5), extravasation (*n* = 10), and medication education (*n* = 133). Questions related to specific treatments like ‘Cycle 1 Follow up’ (*n* = 862), ‘Post recommencement of SACT’ (*n* = 5), and ‘Treatment Reaction’ (*n* = 25) also fall under this category.
- Post-treatment follow-up and review: This includes cases of post-discharge follow-up (*n* = 209), general follow-up (*n* = 197), shared care review (*n* = 421), and post-SURC presentation (*n* = 95). It also includes issues related to care equipment like CVAD (central venous access device) (*n* = 168), 5FU (5-fluorouracil) infuser and central venous access devices (CVAD) (*n* = 12), and PEG (percutaneous endoscopic gastrostomy) (*n* = 13) concerns and problems that required review.
- COVID-related queries: The impact of COVID-19 also surfaces significantly in this data, with specific categories for COVID-related queries (*n* = 156), COVID questions (*n* = 41), and COVID PCR (polymerase chain reaction) (*n* = 31).
- Administrative and logistical inquiries: This category encapsulates questions related to appointment times (*n* = 49), requesting a medical certificate (*n* = 2), scheduling or follow-up on radiology tests (*n* = 74), pathology form inquiries (*n* = 14), and patient admission at outreach sites (*n* = 2).

## Discussion

Early recognition and management of symptoms allow for continued adherence to the planned treatment regimen, ensuring optimal treatment outcomes, patient safety, and maintaining treatment continuity [1, 2]. The dedicated focus on symptom management and support within SURC contributes to improved patient satisfaction and reduced anxiety [2]. This study explores the implementation and evolution of SURC in a large regional oncology setting, where the vast geographical distribution of patients brings in its own challenges and opportunities.

We feel a significant number of patients who presented to ED but not directly admitted to hospital (namely discharged home, admitted to the short-stay unit or discharged home) would be appropriate for the SURC clinic and therefore free up much needed beds in the Emergency Department. Over 800 patients during the study period were admitted to the hospital for an inpatient stay and we would hope with the implementation of the SURC to better support and educate patients throughout treatment, the number of hospital admissions would also decrease. In addition to this, we would ensure patients are prevented from unnecessary pathology and diagnostic testing also saving the health service additional monetary funds. With the SURC model providing access to dedicated oncology nursing and medical staff that are easily contactable through a single point of access, treatment delay and cancellations are more often prevented. The SURC service aims to integrate the Optimal Cancer Care Pathways (OCPs) as well as align with the current 2016–2020 Cancer Action Plan to ensure a strong focus on inclusive services and service accessibility, capacity of individuals and health literacy at the core of cancer service delivery (Cancer Action Plan, 2016–2020) [9].

The nurse-led SURC model has been reported to achieve an investment return of $1.73 for every dollar invested [4]. Beyond the economic benefit of SURC, there are direct tangible benefits to the patients as well as intangible benefits to all the stakeholders of SURC. Our findings of month-on-month increase in patient contact since implementation of the model, range of clinical activities that accounted for the episodes of care, reduced ED presentations, and providing a direct hospital admission pathway are consistent with what has been previously reported. This component of the SURC model addresses the current gap in the healthcare system where patients who are expected to experience disease and treatment-related symptoms are given an alternate avenue to timely access to expert advice and care. The majority (79%) of encounters were via telephone, which, in the absence of SURC, would have either resulted in ED presentations or delayed reporting/presentations/further complications due to lack of access to medical service in a timely manner. With the high volume of presentations from 2020 to 2023, we observed a significant increase in SURC adoption, reflecting patients and healthcare providers’ growing confidence in this model. This trend is aligned with previous studies supporting the need for patient-centred care models in oncology [10]. Our results add to the literature, reinforcing SURC as an effective patient-centred approach.

Beyond the established model of care of SURC, to adapt to regional healthcare challenges and the evolving pandemic situation during the study period, the model was modified at BRICC. This included shared care of oncology patients (alternating pre-treatment patient reviews with the treating oncology team, on an agreed list of treatment regimens of low complexities), delivering front-line COVID-19 response to oncology patients, and pre-emptive post-cycle 1 day 4 and 8 contact to assess tolerance and toxicities of SACT. This proactive contact with patients post-cycle 1 SACT is clinically meaningful, as it provides an opportunity to assess patient tolerance to treatment and anticipate clinical issues that could then be addressed at an early stage. This reduces treatment delays and cancellations, potentially leading to better health outcomes and resource utilisation. Clinician and patient feedback highlighted increased confidence in the service, reduced workload for the chemotherapy day unit and overall satisfaction for the service.

## Limitations

As a single-centre study, our results may not be generalisable to all healthcare settings, particularly those with different patient demographics, organisational structures, or resources. Consequently, potential biases might have influenced our findings. Firstly, a selection bias could have occurred since patients who presented at SURC are likely to have been those more comfortable with this model of care, possibly skewing the data towards a positive outcome. Additionally, the study’s reliance on historical and observational data might have led to an information bias, with potential inaccuracies or inconsistencies in record-keeping impacting the study’s validity. It is also worth noting that part of the data was obtained during the COVID-19 pandemic, a time of significant disturbance and adjustment for healthcare systems worldwide.

## Conclusion

This study demonstrated the benefit of a dedicated acute service for patients with cancer living in a regional area for management of symptoms as well as providing a dedicated assessment early in their treatment course. It also provided a unique insight into looking after patients with cancer during the COVID-19 pandemic.

## Data Availability

The de-identified data we analysed are not publicly available, but requests to the corresponding author for the data will be considered on a case-by-case basis. All authors had full access to all of the data related to the study.
